# Pea cultivar Blauwschokker for the phytostimulation of biodiesel degradation in agricultural soil

**DOI:** 10.1007/s11356-019-06347-9

**Published:** 2019-10-24

**Authors:** Małgorzata Hawrot-Paw, Patryk Ratomski, Małgorzata Mikiciuk, Jacek Staniewski, Adam Koniuszy, Piotr Ptak, Wojciech Golimowski

**Affiliations:** 1 Department of Renewable Energy Sources Engineering, Papieża Pawła VI 1, 71-459 Szczecin, Poland; 2 Department of Plant Physiology and Biochemistry, West Pomeranian University of Technology, Słowackiego 17, 71-434 Szczecin, Poland; 3 Institute of Chemical Technology and Engineering, Poznan University of Technology, Berdychowo 4, 60-965 Poznań, Poland; 4 Department of Agroengineering and Quality Analysis, Wrocław University of Economics, Komandorska 180/120, 53-345 Wrocław, Poland

**Keywords:** Biodiesel, Phytostimulation, Microorganisms, Soil, Pea cultivar Blauwschokker

## Abstract

Phytoremediation is a cost-effective and ecologically friendly process that involves the use of plants to uptake, accumulate, translocate, stabilize, or degrade pollutants. The present study was conducted to demonstrate the potential of pea (*Pisum sativum* L. spp. *sativum*) cultivar Blauwschokker to phytostimulate biodiesel degradation in an agricultural soil, considering the influence of biological remediation on selected physiological parameters of plants and the amount and activity of soil microflora. Biodiesel was spiked into soil in dose of 50 g kg^−1^ of dry mass soil. The results of the study showed that the rate of biodiesel degradation in the vegetated soil was higher than that occurring by natural attenuation. At the same time, biodiesel showed a positive effect on the growth, development, and activity of soil bacteria and fungi. Moreover, the obtained results showed an improvement in physiological parameters of plants, including an increase in chlorophyll *a* and total chlorophyll content and higher relative water content in leaves in the presence of biodiesel.

## Introduction

Crude oil and its derivatives may accumulate in the environment not only during their exploration but also during the storage, transport, and combustion of fuels. The components of these fuels, mainly petroleum hydrocarbons, pollute soil, water, and air and also have an adverse impact on plants, animals, and humans. Several environmental and economic aspects have contributed to the increased interest in the production of biofuels which are alternative, renewable, and less toxic fuels compared to conventional fuels. An alternative to diesel fuel is biodiesel which is used as a fuel or as a biocomponent of conventional fuels.

For many years, biodiesel has been considered as a nearly perfect source of energy, largely due to environmental reasons and the possibility of its production from renewable substrates. Biofuels have been considered as highly biodegradable substances and enabled reducing emissions of toxic components of exhaust gas (Lai 2014; Jedynska et al. 2015). However, several studies have indicated that biodiesel may have an adverse impact on the environment, including physicochemical and biological properties of soils (Singh et al. 2014; Hawrot-Paw et al. 2017). This impact may result from the chemical composition of biodiesel that, inter alia, includes some toxic substances that aim to prevent the oxidation of biodiesel (Tamada et al. 2012). In addition, the biodiesel-contaminated soil may contain toxic methanol, a product of reverse transesterification reaction (Leite et al. 2011; da Cruz et al. 2012). Moreover, the presence of fuels in soil, including environmentally friendly biofuels, leads to a loss of the properties of soil. Therefore, it is primarily essential to curb the spread of these pollutants in the environment by using physical, hydraulic, permeable, and active barriers and to immobilize the contaminants. Subsequently, light organic liquids must be collected from the groundwater table, and then remediation processes be conducted both at the site of contamination (in situ) and away from it (ex situ) (Etim 2012; Azubuike et al. 2016). The alternatives to expensive chemical and physical techniques are biological methods which are cheaper and less environmentally intrusive. The advantages of biological methods are reflected in the biological remediation, including biodegradation processes, that uses the activity of soil microorganisms to decompose contaminants, as well as phytoremediation that uses the potential of plants to this extent. Depending on the type of contaminants and their potential to undergo degradation, several phytoremediation mechanisms may be utilized, including phytoextraction—the absorption and accumulation of contaminants from soil to roots and sprouts (Denise et al. 2013), rhizofiltration—the use of roots of plants to remove contaminants from waste water (Akpor et al. 2014), phytostabilization—the reduction of the spread of soil contaminants (Montenegro et al. 2016), phytotransformation—the absorption and degradation of contaminants by plants (Lotfinasabasl et al. 2013), and phytostimulation—the stimulation of microbiological degradation in the rhizosphere zone (Khatoon et al. 2017), including stimulating the activity of microorganisms that degrade oil hydrocarbons (Agamuthu et al. 2010). The effect of the phytoremediation technology depends on the depth of the plant root system and the level of contamination, which is generally relatively low. The efficiency of this technology also depends on the type of soil and plant, the microbiological activity of the soil, and the interaction between these factors (Hajabbasi 2016). A vast number of studies on the phytoremediation of petroleum-based hydrocarbons have shown the high potential of various species of grasses and legumes in the degradation of contaminants (Hall et al. 2011; Basumatary et al. 2012; Bramley-Alves et al. 2014).

Phytoremediation is a very promising ecological method employed to clean up different matrices from different contaminants (Di Gregorio et al. 2014, 2015; Matsodoum Nguemté et al. 2018). A number of studies thus far have focused on the phytoremediation of diesel fuel-contaminated soil (Dadrasnia and Agamuthu 2013; Olajuyigbe and Aruwajoye 2014; Hatami et al. 2018); however, relatively little is known about the phytoremediation of soil contaminated with biodiesel, and it still remains unclear if phytoremediation technology is appropriate for removing biofuel from soil. One of the challenges of phytoremediation is identifying appropriate plants for degradation of pollutants. Some studies have demonstrated the potential of ground soybeans, sunflower seeds, Lord-variety yellow lupine, and Eureka-variety field pea in the remediation of biodiesel (Hawrot-Paw et al. 2015).

This study investigates the ability of pea cultivar Blauwschokker to promote the degradation of biodiesel fuel in the soil and the relationship between the soil pollution and the reaction of soil microflora and plants. The assessment of the phytostimulation effect was conducted under controlled experimental conditions.

## Materials and methods

### Soil

The soil used in this research was collected from the Agricultural Test Station of the West Pomeranian University of Technology in Szczecin, located in Lipnik near Stargard in Poland (53°20′N, 14°58′E). The soil was a fraction of clay sand (Table 1) and was taken from a depth of 0–15 cm of the arable-humic horizon. Following collection, the soil material was dried and then passed through a sieve with a mesh size of 2 mm.

**Table 1:** Physiochemical properties of soil

Soil texture equivalent diameter size and percentage of fraction			C	N	pH_KCl_
Sand 2.0–0.05 mm	Silt 0.05–0.002 mm	Clay < 0.002 mm	g kg^−1^ DM soil		
79.7	18.9	1.4	10	1.1	6.80

### Plant

The test plant used in this study was pea (*Pisum sativum* L. spp. *sativum*) cultivar Blauwschokker. This plant was selected based on the results of a separate experiment (not published), in which the germination index was determined according to the characteristics of seed germination and the elongation of roots was measured from top to bottom of the hypocotyl base in the presence of biodiesel introduced into soils at doses of 10 and 50 mg g^−1^ DM soil (loamy sand and sandy loam). Twenty-four plants (15 species), taxonomically representing six families (*Fabaceae*, *Poaceae*, *Boraginaceae*, *Rosaceae*, *Brassicaceae*, *Asteraceae*), were used in that experiment. The pea cultivar Blauwschokker was the only plant that showed resistance to the diesel and biodiesel fuels present in the soil, regardless of the soil type and the dose of contamination. The values of germination index and root length of seedlings grown in the biodiesel-contaminated soils were found to be higher compared to control.

**Fig. 1:**
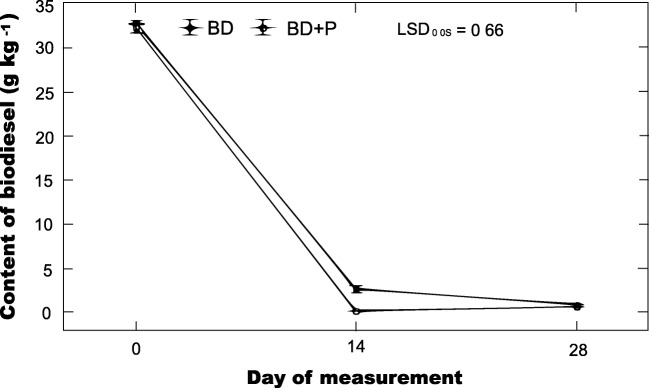
Dynamics of changes in the content of biodiesel in soil during incubation

### Chemical samples and analysis

Biodiesel used in this research was provided by one of the Polish refineries. It was composed of 96.5% (m/m) of fatty acid methyl esters, which is in compliance with the Polish and European standards. The density of biodiesel ranged from 860 to 900 kg m^−3^, and the kinematic viscosity at 40 °C was 3.5–5.00 mm^2^ s^−1^. Biodiesel is a flammable liquid with an intense yellow color (www.orlen.pl). The content of biodiesel in the soil sample was determined using a Hewlett-Packard 6890 gas chromatograph coupled with a flame ionization detector and an automated injection tower (model 7683). Phenomenex’s ZB-1 HT Inferno column (30 m × 0.25 mm × 0.25 μm) with a pre-column (5 m × 0.32 mm) was employed in the chromatographic analysis. The carrier gas used was hydrogen which was fed at a constant flow rate of 2.1 cm^3^ min^−1^. All the determinations were made at a programmed temperature: an initial temperature of 50 °C for 2 min, which was then increased at 25 °C/min to 300 °C for 5 min. Injections were made in splitless mode at 250 °C. Octadecane (C18) for gas chromatography (Merck) was used as a standard at a concentration of 1 μg/μl in hexane. In addition, a standard solution of biodiesel oil was prepared in hexane at a concentration of 5 μg/μl. Both the standard solutions were placed in Certan® vials and stored in a refrigerator. The extraction efficiency was determined for soil samples containing a known amount of biodiesel oil. Biodiesel oil in standard samples and biodiesel residues in the investigated soil samples were extracted using 95% Baker hexane for high-performance liquid chromatography (10 cm^3^ of hexane per 1 g of soil). On completion of the extraction, 8 μl of the hexane phase was withdrawn and then added to 1 cm^3^ of hexane containing 23 μl of a standard C18 solution. The obtained samples were dried with anhydrous magnesium sulfate and analyzed by gas chromatography under the aforesaid conditions. The extraction efficiency in standard samples was estimated to be 98.3 ± 1.5%.

### Experimental design and biological analysis

To estimate the current moisture content of the soil, dry-oven method was employed. Based on the result obtained, the moisture content of the soil was brought to 50% of the maximum water holding capacity. The soil material was divided into two parts: one was left without additives (control—treatment C), and the other was mixed with biodiesel at a dose of 50 g kg^−1^ of dry mass of soil (treatment BD). Another factor tested in the experiment was the effect of the presence of plants (P). To evaluate this factor, four research combinations were created: C, P, BD, and BD + P. For each variant, soil material weighing 2000 g was placed in a pot with a capacity of 2500 g. Per treatment were assigned four pots which were arranged in a completely randomized block design. Commercial fertilizers—ammonium nitrate (250 kg N ha^−1^) and superfosfat (120 kg P_2_O_5_ ha^−1^)—were added per 20-cm soil layer to all the pots. Ten pea seeds were sown at a depth of 1.5 cm of soil in these pots. Following emergence, seedlings were thinned to five per pot. Plants were grown in greenhouse microcosms. A SON-T AGRO sodium lamp with a photoperiod of 16 days/eight nights was used for illumination. The experiment was conducted at a temperature of 21 ± 1 °C. The moisture content of the soil was monitored and maintained at a constant level throughout the experiment.

On the first, 14th, 28th, and 56th day of the experiment, the content of biodiesel in the soil and the number of microorganisms and microbial respiration were determined. For this analysis, the soil samples were collected using a stainless steel soil probe sampler. For chemical and microbial analysis, the soils were taken in triplicates from the full depth or from the plant rhizosphere zone in each pot (approx. 60 g) and mixed thoroughly. Physiological features of pea (content of photosynthetic pigments, relative water content (RWC), and photochemical activity of photosystem II (PS II)) were tested on the last day of the experiment.

The number of microorganisms in the soil was estimated using the soil culture dilution method. This estimation was carried out using appropriate culture media for five groups of microorganisms: bacteria—Bunt and Rovira medium (1955), actinobacteria—Cyganov and Žukov medium (1964), fungi—medium with streptomycin and rose bengal according to Martin (1950), biodiesel-degrading microorganisms—modified Bushnell–Haas medium (1941), and heterotrophic microorganisms—nutrient agar. For the analysis, the test material was taken from the plant rhizosphere zone from each pot. Microorganisms were cultured at a temperature suitable for each type of organisms (25 °C for bacteria (3 days), fungi (5 days), and actinobacteria (7 days) and 28 °C for heterotrophs (2 days) and biodiesel-decomposing microorganisms (7 days). The numbers of microorganisms were determined in three replicates. The results (average taken from four dates of analysis) were presented as colonies forming units (CFU) per 1 g of dry mass of soil (CFU g^−1^ DM soil). The microbial respiration in the soil was determined according to PN-EN ISO 16072:2011. This analysis was performed in fresh soil using an NaOH solution that had absorbed CO_2_ emitted by soil microorganisms.

In this study, the following physiological features of plants were evaluated: water balance, content of assimilation pigments in leaves (chlorophyll *a*, chlorophyll *b*, total chlorophyll), and the parameters of chlorophyll *a* fluorescence induction. The content of chlorophyll *a*, chlorophyll *b*, and total chlorophyll in the leaves was determined in three repetitions using the method of Arnon et al. (1956) with modification described by Lichtenthaler and Wellburn (1983). Assimilation pigments were extracted from the leaves by grinding the samples of fresh matter collected from representative leaves, weighing about 0.05 g, in a mortar with 10 cm^3^ of 80% acetone. Homogenates were then centrifuged for 10 min at 1500 rpm. The optical density of the samples was determined spectrophotometrically at the wavelengths of *λ* = 440, 645, and 663 nm, using a spectrophotometer (Marcel Mini). The water balance was calculated according to the method of Bandurska (1991) in three repetitions based on the following indices: RWC—the index that specifies the ratio of the amount of water in the leaf tissue to the amount of water in the fully saturated plant, and water saturation deficit (WSD) in the leaf tissue—the index that specifies the ratio of the amount of water deficit in the leaf tissue to its maximum saturation. The chlorophyll fluorescence parameters were recorded using a Handy PEA (Hansatech) spectrofluorometer according to the standard procedure of the manufacturer (3 nm × 650 nm LEDs, maximum actinic light intensity 3000 μmol m^−2^ s^−1^). The measurements were made on ten representative leaves from each experimental variant, which were the first, fully developed and compound leaves counting from the top of the plant. The leaves were shaded 20 min before being measured with factory clips (illuminated area was 4 mm in diameter). The following parameters of chlorophyll fluorescence induction were measured and calculated using the spectrofluorometer: F_0_—initial fluorescence (zero) (excitation energy loss index in power antennas), F_M_—maximum fluorescence after reduction of acceptors in PS II and after adaptation to dark, F_V_ = F_M_ − F_0_—variable fluorescence determined after adaptation to dark (the parameter dependent on the maximum quantum yield of PS II), F_V_/F_M_—maximum potential photochemical reaction efficiency in PS II determined after adaptation to dark and after reduction of acceptors in PS II, T_FM_—time of chlorophyll fluorescence increase from the beginning of measurement to reaching the maximum (F_M_), PI—PS II vitality indicator that concerns the general vitality of this system, am (Area)—surface above the chlorophyll *a* fluorescence induction curve between the F_0_ and F_M_ points proportional to the pool size of the reduced plastoquinone acceptors in PS II (Kalaji and Łoboda 2007).

### Statistical analysis

The results of this research were statistically analyzed using the computer program STATISTICA, version 13.1, developed by StatSoft Poland. A multifactorial analysis of differences was carried out to assess the influence of biodiesel, the presence of plants, and the date of analyses on the selected microbiological parameters, whereas a single-factor analysis was carried out for evaluating the physiological features of plants. The significance of differences between the averages was determined by Duncan’s test at a level of *α* ≤ 0.05.

## Results and discussion

Changes in the concentration of biodiesel in the soil are depicted in Fig. 1. The initial content of biofuel determined using chromatographic methods was approximately 33 g kg^−1^. The biodiesel depletion in absence of pea plants was significant, 91% in 14 days. However, the amount of depletion of biodiesel in the soil vegetated with pea plants was significantly higher, 99% after 14 days of incubation. On the 28th day of experiment, the content of biodiesel in unplanted soil was 0.9 g kg^−1^, while in vegetated soil the concentration was over 20% less. The roots penetrate the soil and bulk soil was transformed into rhizosphere soil with associated active microbial community (Kamath et al. 2004). That plants enhance the rate of degradation of conventional diesel fuel has been already observed; however, biodiesel has a different chemical composition, which may cause differences in its susceptibility to biological degradation (Junior et al. 2009). The efficiency of phyto-based technology does not only depend on the type of fuel and the level of contamination. Many other factors also play a role, such as physiochemical parameters of soil, and nutrient and water availability for plants (Jagtap et al. 2014; Magdziak et al. 2014). However, the most important aspect is the capacity of plant to growth in presence on contaminants. The ability of plants to grow in contaminated soil largely depended on the plant species. Most of the plants are not resistant to fuel presence in the soil (Hawrot-Paw et al. 2015). This is confirmed by the research carried out by Hawrot-Paw and Hreczuk (2009) which evaluated the germination potential of 14 plant species and their varieties on diesel oil and its blend with biodiesel (diesel oil 95%:biodiesel 5%) at a dose of 50 g kg^−1^ of dry mass of soil. For more than half of the analyzed plants, the germination index in the presence of biodiesel was found to be reduced compared to its value in the presence of conventional fuel. The results of our previous screening experiment indicated that more than 50 plant species (including crop plants) showed a significant decrease in germination index in the biodiesel-contaminated soil, while the pea cultivar Blauwschokker alone was resistant to its presence (data not shown). *Fabaceae* species are able to form symbiosis with various beneficial microorganisms. That symbioses provide nutrients for the plant and stimulate their growth (Safronova et al. 2011). Fuels may prevent or reduce water and oxygen from entering the seeds (Zarinkamar et al. 2013) but the germination index of pea seeds in the biodiesel-contaminated soils was higher compared to control. In this context, it is worth mentioning that in this study pea, plants have significant effect on the degradation of biodiesel in comparison to unplanted soil. The biodiesel may be degraded in the rhizosphere by root-released pea plant enzyme (Ioannis 2015) or by stimulation of microbial activity that leads to degradation of contaminants to nontoxic or harmless products (Chandra and Kumar 2017). Plants exude compounds that increase the bioavailability of contaminants (Zand and Hoveidi 2016), which affects the level of degradation in the soils (Shukla et al. 2013). It is known that *Fabaceae* species enhance the bioavailability of conventional fuel for microbial degradation (Hall et al. 2011). Even though further research is necessary to explain the mechanisms, data here obtained showed that pea plants germinate and grow in the presence of biodiesel and stimulate the degradation of biofuel.

**Fig. 2:**
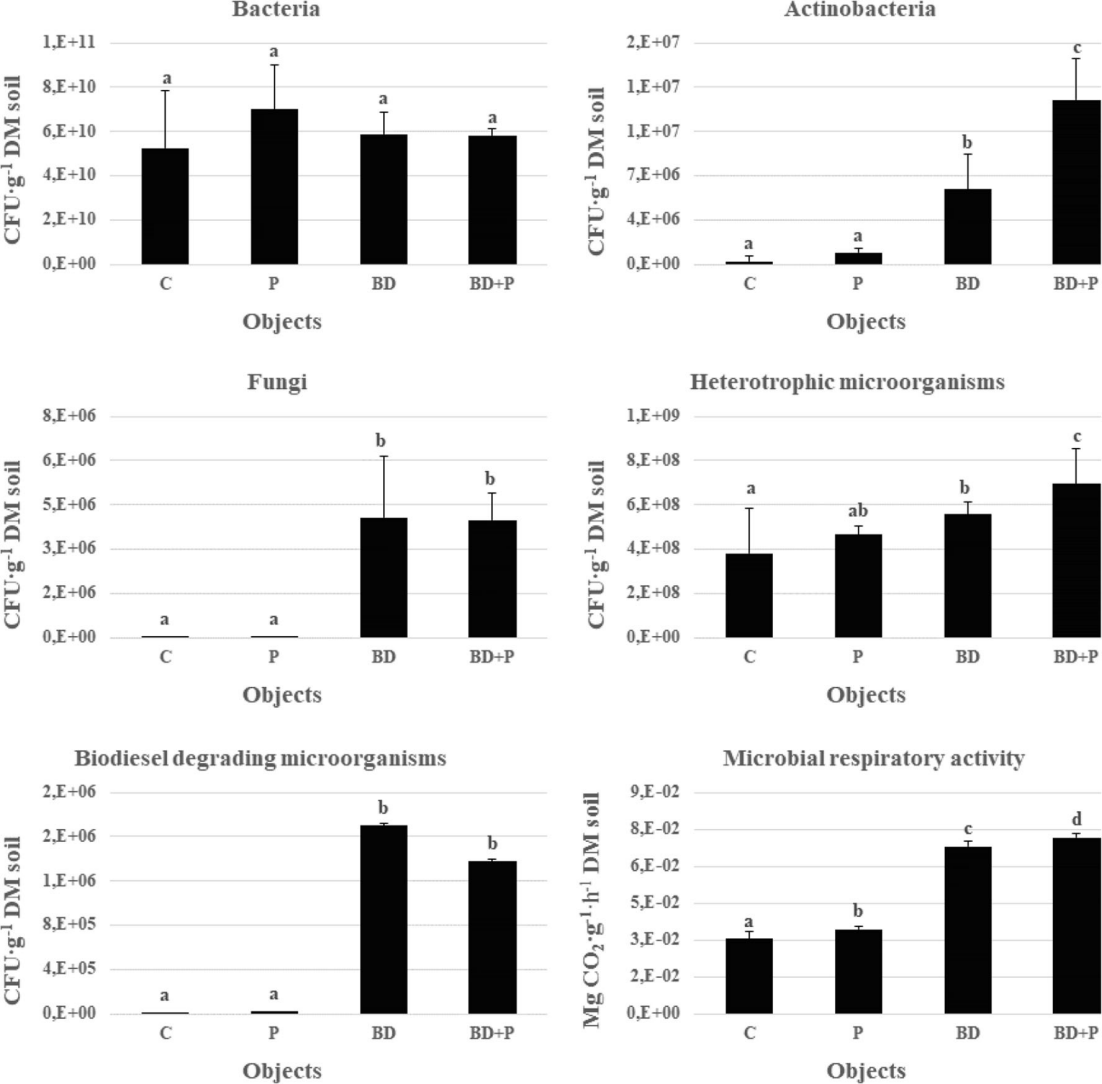
Average number of microorganisms and microbial respiration in individual variants of the experiment (mean over each column not marked with the same letter is significantly different at *P* < 0.05)

This study showed that the introduction of biodiesel into the soil had a significant effect on the number of microorganisms and microbial respiration (Fig. 2). According to Lapinskiene et al. (2006), the conventional fuels in the soil may serve as a source of energy for microorganisms and may promote their growth, according to Hawrot-Paw (2012), the impact of oil derivatives in the soil on the growth of microorganisms is ambiguous—similar is the case with the impact of biofuels. In this study, biodiesel was found to stimulate the growth and development of bacteria, which was consistent with the results of the research of Hawrot-Paw and Izwikow (2015). The average number of bacteria in the biofuel-contaminated soil was higher compared to the control variant, but this difference was not statistically significant. Biodiesel also showed a positive influence on the number of actinobacteria. Values marked in the BD variant (5.93 × 10^6^ CFU g^−1^ DM soil) were over 2000% higher than the values in the uncontaminated soil. A significant increase was also observed in the number of fungi. The average number of fungi in control (8.81 × 10^3^ CFU g^−1^ DM soil) and biodiesel-contaminated soils (4.07 × 10^6^ CFU g^−1^ DM soil) indicated the stimulating effect of biofuel. Similar results were observed in the previous studies concerning the biological activity of the soil contaminated with diesel oil and biodiesel (Hawrot-Paw and Izwikow 2015). The number of bacteria, actinobacteria, and fungi in planted soil in this study was higher compared to the control. Plants release a variety of organic compounds in the soil which can be used as carbon sources by heterotrophic fungi and bacteria (Zand and Hoveidi 2016). Plant roots stimulate microbial consortia of rhizosphere by aerating the soil and also providing surface for colonization (Prabhu et al. 2017). Legumes provide stimulation of biodiesel degradation, which may be related to root secretions rich in nitrogen compounds (Fustec et al. 2010).

A number of bacteria and fungi in the plant–bacteria–fungi association have been found to be involved in the degradation of contaminants (Bell et al. 2014; Ozyigit and Dogan 2015). More in particular, results obtained suggest that biodiesel mainly promotes the development of actinobacteria during the vegetative growth of plants. In this context, it is worth mentioning that actinobacteria play major roles in the cycling of organic matter, inhibit the growth of several plant pathogens, improve the availability of nutrients, enhance the production of metabolites, and produce plant growth regulators (Bhatti et al. 2017). Vegetation of the soil with pea plants increases the number of actinobacteria that positively correlates with biodiesel degradation. An evident competition between fungi and actinobacteria is not evident. Both fungi and actinobacteria play important functions in soil microbiome and both are described as good candidates in the degradation of pollutants such as petroleum hydrocarbons or PCBs (Siracusa et al. 2017; Nzila et al. 2018; Becarelli et al. 2019).

Moreover, more in general, the presence of biofuel was found to have a positive influence on heterotrophic microorganisms; the average number of heterotrophs in the treatment variants was almost 50% higher compared to the control variant. However, the reason behind the positive impact of biodiesel on this group of microorganisms was not clear. In a previous study on the relationship between soil contamination with diesel and biodiesel fuel and the reaction of soil microflora (Hawrot-Paw et al. 2015), a reduction in the number of heterotrophic microorganisms was noted after 43 days of the experiment. Plants can stimulate microbiological activity of soils, and a number of researches conducted on soils contaminated with conventional fuels have proven the positive impact of plants belonging to *Fabaceae* family on microorganisms (Merkl et al. 2005; Liste and Felgentreu 2006; Diab 2008). In this study, the pea cultivar Blauwschokker was found to have a stimulating effect of on microorganisms. The average number of heterotrophic microorganisms (6.96 × 10^8^ CFU g^−1^ DM soil) in the BD + P variant was 36% more than in the BD variant. In the experiments conducted by Hawrot-Paw et al. (2015) in the plant rhizosphere zone of the pea cultivar Eureka, more than 30% stimulation of heterotrophic microorganisms and more than 85% growth of microbes decomposing diesel oil were observed. Biodiesel primarily consists of fatty acid esters, which are also synthesized in the natural environment (Lapinskiene and Martinkus 2007) and may have a positive effect on the number of microorganisms and microbial respiration, including those involved in its biological degradation processes. In this study, the number of microorganisms that degrade biodiesel was found suitable for the biological decomposition of conventional fuels. The average number of biodiesel-decomposing microorganisms was higher than in the control soil—1.7 × 10^6^ CFU g^−1^ DM soil in the BD variants and 1.38 × 10^6^ CFU g^−1^ DM soil in the P variants. For the biodegradation process, microbial activity is more important than the number of microorganisms, and it was higher in the presence of plants, suggesting a pea plant phytostimulation effect. Root exudates or rhizodeposits can increase contaminant bioavailability for soil microbes (Azaizeh et al. 2011) and effectiveness of the degradation.

In this study, the highest microbial respiration, approximately 130% more, was recorded in the planted soil (BD + P variant) than in the control variant. These plant–microbe interactions have been successfully exploited to alleviate toxicity by decreasing the concentration of pollutants in the rhizosphere (Ozyigit and Dogan 2015).

This study found that biodiesel had a favorable effect or did not have any adverse impact on most of the analyzed physiological parameters of the pea, which signifies a high level of tolerance of the cultivar to the high level of soil contamination. In the present research, the plants growing in the soil contaminated with biodiesel (BD + P variant) were characterized by higher values of relative water content (RWC) and lower values of deficit of water saturation (WSD) in tissues, as well as a higher content of chlorophyll *a* and total chlorophyll in leaves, compared to the plants from the P variant. The contamination of soil with biodiesel, however, had no effect on the content of chlorophyll *b* (Table 2). The research conducted by Hawrot-Paw et al. (2015) on the pea plant cultivar Eureka for revealed that the contamination of soil with biodiesel reduced the RWC but had no effect on the content of chlorophyll *a*, chlorophyll *b*, and total chlorophyll in the leaves of the plant. For a number of plant species, numerous authors have pointed out that the contamination of soil with petroleum-based compounds resulted in an adverse impact on the synthesis of assimilation pigments by their negative effect on physical and chemical properties of the soil, which are preconditions for the availability of plant nutrients (Odjegba and Sadiq 2002; Adenipekun et al. 2008; Balliana et al. 2017).

**Table 2:** Values of physiological parameters of pea cultivar Blauwschokker growing in the soil contaminated with biodiesel

Object	RWC	WSD	Content of chlorophyll *a* in leaves	Content of chlorophyll *b* in leaves	Content of total chlorophyll in leaves	F_V_/F_M_	T_FM_ (ms)	PI	Area (bms)
	[%]		[mg g^−1^fresh matter]						
P	66.93^a*^ ± 4.54	33.07^b^ ± 4.54	1.33^a^ ± 0.18	0.70^a^ ± 0.10	2.03^a^ ± 0.28	0.648^a^ ± 0.068	324.00^b^ ±53.79	0.254^a^ ± 0.091	15,378^a^ ± 3638
BD+P	83.73^b^ ± 2.16	16.27^a^ ± 2.16	1.94^b^ ± 0.03	0.78^a^ ± 0.02	2.72^b^ ± 0.05	0.640^a^ ± 0.049	303.00^a^ ± 34.66	0.200^a^ ± 0.086	12,915^a^ ± 2670

*Mean over each column not marked with the same letter is significantly different at *P* < 0.05

*RWC* relative water content in leaves, *WSD* water saturation deficit in leaf tissue, *F*_*V*_*/F*_*M*_ maximal potential efficiency of photochemical reaction in PSII determined after darkening adaptation, *T*_*FM*_ chlorophyll fluorescence growth time from the beginning of the measurement to the maximum, *PI* PSII photosystem vitality indicator, *Area* surface above the chlorophyll *a* fluorescence induction curve between the F_0_ and F_M_ points proportional to the pool size of the reduced plastoquinone acceptors in PS II

A method for determining the efficiency of the plant photosynthetic apparatus that is particularly susceptible to stress factors and for evaluating the reactions of plants to those factors and the effectiveness of their repair mechanisms is the analysis of the chlorophyll *a* fluorescence signal (Van der Tol et al. 2009). In this study, no differences were found in the values of F_V_/F_M_ and PI parameters between the experimental variants (P and BD + P). The parameters F_V_/F_M_ and PI were characterized by low values, indicating the earlier influence of stress factors on the tested plants, which damaged the functions of PS II, thereby reducing the efficiency of electron transport. According to Kalaji and Łoboda (2010), the ratio F_V_/F_M_ is not always correlated with the content of chlorophyll in leaves. This result was confirmed in the present study in which a high content of all forms of chlorophyll was found in leaves.

It was found that the addition of biodiesel resulted in shortening of the growth time of chlorophyll fluorescence (T_FM_) from the beginning of the measurement to the maximum. The values of the T_FM_ parameter characterizing the condition of the photosynthetic apparatus of the analyzed pea variety, regardless of the variant of the experiment, should be considered low, and according to Lichtenthaler et al. (2004), the values are usually in the range of 500–800 ms. The soil contamination did not affect both the indicator of value of PS II vitality index, describing its general vitality, and on the amount of reduced electron acceptors in PS II (Area), which is one of the best performance indicators of the photosynthetic apparatus of the plant (Table 2).

There are candidates (bacteria, fungi) capable of degradation of biodiesel in the soil but this biofuel is relatively resistant to the activity of natural microflora. Moreover, decomposition of the biodiesel is slower than the petroleum-based diesel (Hawrot-Paw et al. 2011). Plants can help solve this problem. The plant rhizosphere-microbe associations can enhance microbial activity and degradation of contaminants. Suitable selection of plants and soil microbes will ensure the depletion of biofuel in a short time.

## Conclusions

The pea plants stimulated decomposition of biodiesel in the soil. The content of biofuel in the BD + P variant after 2 weeks of incubation was only 8% of the value identified in the unplanted soil.

The presence of biodiesel in the soil modified the microbiological properties of the soil. Biodiesel also improved the number and activity of bacteria, actinobacteria, fungi, heterotrophs, and other microorganisms involved in its biological decomposition and led to an increase in the respiratory activity of microorganisms. The pea cultivar Blauwschokker exerted a favorable influence on both the number of microorganisms and the microflora activity of the biodiesel-contaminated soil.

Positive changes in the content of chlorophyll, an increase in the relative water content of pea leaves, and a decrease in the value of deficit of water saturation in the plant tissue were observed during the study. The analysis of parameters of chlorophyll fluorescence induction showed a high level of stress and an adverse effect on the photosystem II function, both in the case of peas growing in the soil contaminated with biodiesel and in the control plants; hence, it was difficult to assess the impact of this factor on the condition of the photosynthetic apparatus of the studied variety.

Pea plants (*Pisum sativum* L. *spp*. *sativum*) cultivar Blauwschokker were here described as capable to speed up the degradation of biodiesel, eventually spilled in soil, even at very high concentration of thousands of part per million. In fact, the content of biofuel in vegetated soil, after only 2 weeks, was only 8% of the value identified in the unplanted soil.
